# Symptomatic Vitreous Opacities: Exploring the Mismatch between Clinical Observation of Vitreous Alterations and Self-Reported Symptoms

**DOI:** 10.3390/jcm13206052

**Published:** 2024-10-11

**Authors:** Giulio Bamonte, Clemente Maria Iodice, Rodolfo Mastropasqua, Elon H. C. Van Dijk, Andrea Appeltans, Maria Vittoria Cicinelli, Matteo Menean, Marcel Ten Tusscher, Stuart W. Harmer, Paola Marolo, Enrico Borrelli, Michele Reibaldi, Georgios D. Panos, Lorenzo Motta

**Affiliations:** 1 Department of Ophthalmology, Brussels University Hospital, Av. du Laerbeek 101, 1090 Jette, Belgium; giuliobamonte@hotmail.com (G.B.); andrea.appeltans@gmail.com (A.A.); mtusscher@hotmail.com (M.T.T.); 2 Department of Ophthalmology, HagaZiekenhuis, Els Borst-Eilersplein 275, 2545 AA The Hague, The Netherlands; 3 Faculty of Medicine and Dentistry, University of Rome “La Sapienza”, 00185 Rome, Italy; 4 Ophthalmology Clinic, Department of Medicine and Science of Ageing, University G. D’Annunzio Chieti-Pescara, 66100 Chieti, Italy; rodolfo.mastropasqua@unich.it; 5 Department of Ophthalmology, Leiden University Medical Center, Albinusdreef 2, 2333 ZA Leiden, The Netherlands; e.h.c.van_dijk@lumc.nl; 6 Department of Ophthalmology, IRCCS San Raffaele Scientific Institute, Via Olgettina, 60, 20132 Milan, Italy; cicinelli.mariavittoria@hsr.it (M.V.C.); menean.matteo@hsr.it (M.M.); 7 School of Medicine, Vita-Salute San Raffaele University, Via Olgettina, 58, 20132 Milan, Italy; 8 Applied Electromagnetics Research Group, Department of Engineering, Computing and Design, College Lane, University of Chichester, West Sussex, Chichester PO19 6PE, UK; s.harmer2@uos.ac.uk; 9 Department of Surgical Sciences, University of Turin, 10124 Tulin, Italy; paola.marolo@unito.it (P.M.); enrico.borrelli@unito.it (E.B.); michele.reibaldi@unito.it (M.R.); 10 Department of Ophthalmology, School of Medicine, Aristotle University of Thessaloniki, 54124 Thessaloniki, Greece; 11 Division of Ophthalmology and Visual Sciences, School of Medicine, University of Nottingham, Nottingham NG72UH, UK; 12 Department of Ophthalmology, School of Medicine, University of Padova, 35121 Padova, Italy; lorenzomotta@hotmail.com

**Keywords:** self-reported symptoms, symptomatic vitreous opacities, visual discomfort, vitreous floaters

## Abstract

**Objectives:** To assess the mismatch between the clinical observation of vitreous alterations and self-reported symptoms in young patients complaining of symptomatic vitreous opacities (SVO). **Methods:** The ophthalmic medical records of young patients presenting primarily with SVO were retrospectively evaluated. Symptoms severity was assessed using a questionnaire. The status of the vitreous body was examined with indirect ophthalmoscopy at the slit-lamp and classified according to an ad hoc severity scale. **Results:** Sixty eyes of thirty otherwise healthy patients (median age: 32.5 (IQR: 29.0–37.0) years old) complaining of SVO (median duration: 38 months; interquartile range: 18–84 months) were enrolled. SVO was rated as severe by 50% of participants, affecting all the activities explored in the questionnaire. Twenty-three patients (76.6%) reported SVO-related depression and/or anxiety, for which eleven patients (36.6%) were or had been using medication. Fifty-eight eyes (96.6%) showed no evidence of (or minimal) vitreous opacity, while two eyes (3.3%) were found to have significant vitreous opacity. No significant inter-gender differences (*p* > 0.05) and no significant differences (*p* > 0.05) were found between the severity of vitreous opacity and patients’ reported symptoms nor with their psychological status and medication use. **Conclusions:** Severe discomfort related to the perception of vitreous floaters exists in young patients whose vitreous gel examination is unremarkable or shows only minor alterations. We believe this discrepancy can be explained by optical anisotropy; significant forward-scattering of light, which results in floater symptoms; and reduced back reflection, which limits the clinical observation.

## 1. Introduction

Floaters, sometimes called myodesopsia (Greek) and muscae volitantes (Latin), are entoptic phenomena that are often perceived by the patient as linear grey shadows with dark spots or nodules that pass through their field of vision with eye movements [1]. Primary vitreous floaters are defined as those arising from structures that are endogenous to the vitreous body, in contrast to those secondary to retinal detachment, vitreous hemorrhage, inflammation, lymphoma, and asteroid hyalomins [2].

The causes of primary floater symptoms are localized changes to the refractive index of the vitreous, usually resulting from aggregation of the ultrafine collagen fibrils that are interspersed throughout the vitreous body and the subsequent liquefaction of the surrounding vitreous [3]. These fibrils are subwavelength in “healthy” vitreous, being typically a few tens of nanometers in diameter. However, when the vitreous undergoes degeneration (through ageing, pathology or trauma), these fibrils can coalesce and give rise to the perception of floaters [4,5].

Subwavelength heterogenous structures, like healthy vitreous, behave as media with a homogenous refractive index provided certain conditions on size and spacing are met. However, with vitreous degeneration and the clumping of vitreous fibrils, the resulting structures no longer provide a homogenous optical environment within the vitreous body, and local refractive index differences between coalesced material and the surrounding vitreous cause the scattering of light propagating from the lens to the retina [3]. In other words, the perception of floaters appears to be due to the diffraction patterns at the retina resulting from such focal refractive index alterations causing optical scattering within the vitreous body [3].

When the vitreous anomalies result in symptoms which disturb the patient, the term symptomatic vitreous opacities (SVO) is often used [3]. SVO were reported to be frequent in elderly phakic patients, and their incidence were found to be even higher following uneventful cataract surgery [6,7].

A diagnosis of SVO is generally straightforward in patients with a posterior vitreous detachment and/or prominent vitreous alterations. On the contrary, young and otherwise healthy patients complaining of SVO often complain of severe disturbances due to vitreous floaters, usually not consistent with the corresponding clinical findings. This mismatch between reported visual discomfort and clinical findings results in a significant underestimation of the problem.

Therefore, the purpose of this study was to investigate the discrepancy between the clinical observation of vitreous alterations and the self-reported symptoms and to analyze their psychophysical impact in a cohort of young patients with SVO.

## 2. Materials and Methods

In this single-center retrospective observational study, the medical records of patients presenting primarily with vitreous-floaters-related symptoms at the Department of Ophthalmology of Brussels University Hospital from September 2018 to October 2019 were evaluated. The current research was performed in compliance with the tenets of the Declaration of Helsinki for research. Informed consent to participate was signed by each participant before the collection of the data. The Institutional Review Board (IRB) of the Brussels University Hospital waived the need for IRB approval for this study as it is retrospective and observational in nature.

The exclusion criteria were secondary causes of vitreous floaters such as uveitis, asteroid hyalosis, vitreous bleeding, recent history of trauma, and best-corrected visual acuity less than 20/25 Snellen equivalent [2,8,9,10]. We also excluded patients with myopia greater than 6D who may be affected by myopic vitreous degeneration [11].

The severity of symptoms associated with SVO, as well as their impact on daily activities, was assessed using an ad hoc questionnaire (Table 1) [12].

Participants were asked to rate their symptoms on a scale from 0 to 3 (0: none; 1: mild; 2: moderate; 3: severe), and to relate their symptoms with a series of common daily activities (i.e., working/studying, having to concentrate on a task, looking at a computer screen, reading a small print, driving, and watching television). Patients were included in the study if they rated their symptoms at least as moderate. Finally, patients were questioned as to whether they would consider themselves as suffering from a form of depression or anxiety and whether were they seeking or had previously sought professional psychological support because of SVO symptoms. Use of any medication during the last 12 months to treat either depression or anxiety because of vitreous floaters was also noted.

All patients underwent comprehensive ophthalmic examination that included best-corrected visual acuity and intraocular pressure measurement using Goldmann applanation tonometry (Haag-Streit, Köniz, Switzerland) [13,14]. Following pupil dilation, vitreous and fundus examination was carried out at the slit-lamp using a 90D lens (Volk Optical Inc., Enterprise Drive, Mentor, OH, USA) by two experienced examiners (GB and AA) in all cases. The assessments made by the two independent examiners were based solely on the documented clinical findings recorded in the patients’ medical records during their initial clinical visits. These records included descriptions from slit-lamp examinations and, where available, imaging results such as fundus photographs or Spectral-Domain Optical Coherence Tomography (SD-OCT) scans. No new direct observations of the patients were conducted as part of this study. The retrospective nature of the data limits our ability to perform a comprehensive or standardized re-evaluation of the vitreous condition beyond what was documented at the time of the initial clinical visit.

The status of the vitreous was classified according to a three-level severity scale: (1) unremarkable vitreous with no evidence of floaters/filaments; (2) normal vitreous but with significant vitreous floaters/filaments; (3) presence of posterior vitreous detachment (PVD). Lacking an internationally agreed upon classification of vitreous opacities, significant vitreous floaters/filaments were defined if vitreous opacities where either abundant in number or if they were (partially) obscuring the view of retina details. A PVD was diagnosed combining ophthalmoscopic findings with Spectral-Domain Optical Coherence Tomography (SD-OCT) B-scans (Heidelberg Engineering, Heidelberg, Germany, using the staging system previously described [15]. In case of disagreement regarding the perceived level of vitreous degeneration, a further assessment was carried out by a third examiner (MtT).

Continuous variables were reported as the median (interquartile range—IQR), and categorical features were reported as the count (frequency). The normal distribution of variables was verified with the Shapiro–Wilk test. Inter-gender differences were explored using the Mann–Whiney U test. Differences in the results of the floaters-related quality of life questionnaire based on the severity of vitreous appearance were explored using the Kruskal–Wallis test, and post hoc comparisons were conducted using Dunn’s method with a Bonferroni correction for multiple tests. Two-tailed statistical significance was defined as *p* < 0.05. All statistical analyses were performed using IBM SPSS software (IBM Corp., Armonk, New York, NY, USA, version 28.0).

## 3. Results

Sixty eyes of thirty otherwise healthy patients (24 males (80%) and 6 females (20%); median age: 32.5 (IQR: 29.0–37.0) years old) presenting to our clinic with SVO were included. Concordance between the independent examiners was achieved in 100% cases and was based on the documented clinical findings; however, the retrospective nature of these assessments may have introduced a degree of variability that could have been minimized in a prospective study with standardized diagnostic imaging. All eyes presented with unremarkable anterior and posterior segments. Snellen best-corrected visual acuity was 20/20 in all cases. The median refractive error was −1.0 D (IQR: −3.0–0.0). Fifty-eight eyes (96.6%), including the two previously vitrectomized eyes and the seven previously YAG-treated eyes, were classified to be on level 1 of the severity scale. Two eyes (3.3%, one right eye and one left eye of two different patients) were found to have significant vitreous floaters in the setting of an otherwise healthy vitreous (level 3 of the severity scale). No eyes had PVD.

Median floaters symptoms duration was 38 months (interquartile range: 18–84 months). Three patients (10.0%) reported that the symptoms were present in 1 eye only, while the rest 27 patients (90.0%) referred bilateral symptoms. Seven patients (23.3%) had undergone Nd-YAG laser vitreolysis in one eye, and two patients (6.6%) underwent vitrectomy for floaters in one eye (elsewhere). All patients who had undergone Nd-YAG laser vitreolysis were not satisfied with the treatment and were still complaining of SVO in the treated eye. One patient who had previously undergone “core”-only vitrectomy for SVO was also unsatisfied with the outcome of the treatment, while the other one, who had undergone complete vitrectomy with PVD induction, was symptoms free in the treated eye but complained of SVO in the contralateral.

The results of the questionnaire regarding symptoms’ impact on subjects’ daily activities are shown in Table 2. SVO were rated as severe by 50% of participants and reported to affect all the activities mentioned in the questionnaire, particularly looking at a computer screen, driving, and having to concentrate on a task. As many as 23 out of 30 patients (76.6%) described themselves as suffering from depression and/or anxiety related to SVO. Thirteen subjects (43.3%) were currently seeking or had previously sought professional psychological support because of symptoms caused by the perception of floaters. Eleven patients (36.6%) were or had been using medication for either depression and/or anxiety caused by floaters during the previous 12 months.

The Mann–Whitney U-Test demonstrated no significant inter-gender differences (*p* > 0.05), and the Kruskal–Wallis test indicated that no significant differences (*p* > 0.05) were found between the severity of vitreous opacity and patients’ reported symptoms, nor with their psychological status and medication use or within genders (Table 3 and Table 4).

## 4. Discussion

The current study aimed to investigate the discrepancy between the clinical observation of vitreous alterations and the self-reported SVO in a cohort of young patients. The choice of focusing on patients aged ≤45 was based on recent studies reporting particular bothersome SVO prevalence in younger, otherwise-healthy individuals [16,17]. Indeed, according to Webb et al., within people largely under the age of 50 years, up to 76% of smartphone users reported experiencing floaters, with 33% claiming to be bothersome [16]. Another study reported that for patients under the age of 55 years, the effect of SVO is so severe that they are willing to accept a 7% risk of blindness in exchange for floater removal, and that persistent floaters can significantly reduce patients’ self-perception of quality of life [17].

SVO-related symptoms were significant among our study population as these were the primary reason for seeking medical attention. According to our inclusion criteria, patients were enrolled when symptoms were defined as being at least moderate (scoring 2 or more on the severity scale), with half of the participants reporting severe disturbances. Looking at a computer screen, driving, and having to concentrate on a task were among the most affected activities explored in our questionnaire. These findings are consistent with previously reported studies [16,17,18,19,20,21]. In addition, more than two thirds of the cases reported SVO to be associated with, or responsible for, a form of anxiety and/or depression, for which professional counseling and/or medication in the form of antidepressants and/or anxiety remedies had been needed recently. This is also in agreement with past research findings, which have reported that patients with vitreous floaters not only have a decreased quality of life but are also psychologically affected [22]. SVO in our study cohort were longstanding, at least 12 months, with half of the subjects complaining for more than 3 years. This finding supports evidence that neurological adaptation to floaters and/or floater resolution over time does not occur in all patients [17].

Notably, seven patients in our study had previously received YAG laser vitreolysis, but they still had SVO in the treated eyes despite being classified as having level 1 vitreous opacities. Two of the subjects included in the current research had even undergone vitrectomy to remove the bothersome floaters. Interestingly, only the patient who had a complete vitrectomy with PVD induction was symptom free in the treated eye, as opposed to the patient who had a partial “core”-only vitrectomy (without PVD induction). In this direction, it is necessary to highlight that, increasingly, patients are being surgically treated for persistent symptomatic floaters, and several studies have demonstrated excellent efficacy, patient satisfaction, improvements in vision, and a low risk profile for vitrectomy for floaters, or so-called “floaterectomy” [23,24].

However, despite the patients’ reported symptoms, only 2 out of the 60 eyes (3%) included in the present study showed significant vitreous opacities, with the remaining eyes presenting negligible alterations. In this regard, apart from the shadowing effect due to the blockage of light passing through, floaters were also reported to cause light scattering from the edges of the floater, causing stray light, which is hardly measurable during routine clinical examinations [3,25]. Moreover, recent mathematical modeling of vitreous opacity predicted that forward-scattered changes in intensity on the retina are four-to-six orders of magnitude greater than the back-scattered intensity available for clinical examination [3,25]. Indeed, the results of these models would provide a solid scientific explanation as to why patients complaining of floaters may have “normal” vitreous upon ophthalmic examination and why the clinically invisible changes in refractive index may induce sufficient variations on the patient’s retina that may explain such complaints.

To our knowledge, our study is the first to highlight that a significant mismatch exists between the clinical observation of vitreous alterations and self-reported symptoms among SVO patients, which is consistent with the finding that negligible changes in the refractive index of the vitreous may produce significant optical effects at the retina but are virtually invisible clinically [3,25].

The main limitations of the present study are associated with its retrospective, cross-sectional design. Indeed, the causality between floaters and depression/anxiety may also be linked to the fact that patients with tendency towards depression/anxiety could be more prone to notice and be bothered by floaters and not the other way around. In addition, these visual complaints may also correspond to a visual somatization linked to a depression/anxiety disorder. Furthermore, it is important to highlight that the subjective perception of symptoms might have been overreported by the patients, especially by those previously treated with Nd-YAG laser vitreolysis or vitrectomy. Moreover, the exclusion of degenerative myopia based on the dioptric power (i.e., −6D) alone may not be sufficient, since such alterations may occur in eyes with low and moderate myopia as well [26]. Also, ultra-widefield acquisition systems, as well as confocal laser or ultrasound-based quantification of the posterior segment findings, might have been more objective in identifying floaters. The last key limitation of this study is its reliance on retrospective clinical records for the independent assessment of vitreous floaters. Since the two graders based their evaluations on previously recorded data, including written descriptions and imaging when available, their assessments may not reflect the same accuracy or consistency as direct, real-time examinations. This could introduce variability in the classification of vitreous floaters, particularly if the original documentation lacked detail. Furthermore, without access to standardized imaging such as ultra-widefield or confocal laser imaging, the independent evaluation of floaters is inherently limited by the subjective nature of clinical descriptions.

## 5. Conclusions

To summarize, we conclude that severe visual discomfort related to vitreous floaters perception may exist in young patients whose vitreous gel examination is unremarkable or shows only minor modifications. While we believe that vitrectomy should not be performed in all cases of patients complaining of floaters, the current findings indicate that clinicians should go deeper than relying solely on slit-lamp biomicroscopy and OCT findings when evaluating patients complaining SVO. It is imperative to assess subjective complaints and patient personality thoroughly, to meet a patient more than once, and to utilize all available diagnostic methods (i.e., quantitative ultrasonography, contrast sensitivity, and light scattering evaluation) to gauge clinical severity and properly select patients for therapy [12,17,18,24].

Additional research is needed to increase our understanding of why some patients develop SVO compared to others and, consequently, which patients are the best candidates for surgical intervention.

## Figures and Tables

**Table 1:** Floaters-related quality of life questionnaire used in the study.

Floaters-related quality of life questionnaire. Part 1 On a scale from 0 to 3 (0 = none, 1 = mild, 2 = moderate, 3 = severe): How severe would you rate the impact of the vitreous floaters you perceive in general? How severe would you rate the impact of the vitreous floaters you perceive while working/studying? How severe would you rate the impact of the vitreous floaters you perceive while concentrating on a task? How severe would you rate the impact of the vitreous floaters you perceive while reading small prints? How severe would you rate the impact of the vitreous floaters you perceive while using a computer screen? How severe would you rate the impact of the vitreous floaters you perceive while driving? How severe would you rate the impact of the vitreous floaters you perceive while watching television? Part 2 On a scale from 0 to 3 (0 = none; 1 = mild; 2 = moderate; 3 = severe): Are your symptoms related to floaters disturbing enough to limit your ability to enjoy leisure activities such as going out with friends, practicing sports/hobbies or going to the beach? Do you feel depressed and/or anxious about the vitreous floaters you perceive? Are you currently seeking or have previously sought professional psychological support because of floaters-related symptoms? Have you taken any medication for anxiety and/or depression because of floaters-related symptoms, in the last 12 months?

**Table 2:** Percentage of patients’ symptoms on a scale from 0 to 3 (0 = none; 1 = mild; 2 = moderate; 3 = severe), both on a general level and regarding a series of common daily activities: working/studying; having to concentrate on a task; using a computer screen; reading small print; driving; and watching television.

	0 (%)	1 (%)	2 (%)	3 (%)	Median (IQR)
General	0.0%	0.0%	50.0%	50.0%	2.50 (2.0–3.0)
Computer	3.3%	13.3%	30.0%	53.3%	3.0 (2.0–3.0)
Reading	20.0%	16.7%	40.0%	23.3%	2.0 (1.0–2.25)
Television	43.3%	23.3%	23.3%	10.0%	1.0 (0.0–2.0)
Driving	16.7%	26.7%	23.3%	33.3%	2.0 (1.0–3.0)
Work/Study	6.7%	6.7%	16.7%	50.0%	2.0 (1.75–3.0)
Concentration	10.0%	10.0%	40.0%	40.0%	2.0 (2.0–3.0)

**Table 3:** Results of the floaters-related quality of life questionnaire stratified according to the severity of vitreous appearance. No significant differences (*p* > 0.05) were found between the severity of vitreous opacity and patients’ reported symptoms, nor with their psychological status and medication use.

	Status of Vitreous *
General	*p* = 0.55
Computer	*p* = 0.78
Reading	*p* = 0.92
Television	*p* = 0.52
Driving	*p* = 0.71
Work/Study	*p* = 0.76
Concentration	*p* = 0.41
Leisure activities	*p* = 0.38
Depression/Anxiety	*p* = 0.75
Medication	*p* = 0.74
Professional psychological support	*p* = 0.52

* Classified according to a three-level severity scale: (1) unremarkable vitreous with no evidence of floaters/filaments; (2) normal vitreous but with significant vitreous floaters/filaments; (3) presence of posterior vitreous detachment (PVD) (*p*-valued were calculated with the Kruskal–Wallis Test).

**Table 4:** Inter-gender differences.

	Gender (Male/Female) *
General	*p* = 0.43
Computer	*p* = 0.46
Reading	*p* = 0.86
Television	*p* = 0.94
Driving	*p* = 0.63
Work/Study	*p* = 0.27
Concentration	*p* = 0.37
Leisure activities	*p* = 0.37
Depression/Anxiety	*p* = 0.78
Medication	*p* = 0.90
Professional psychological support	*p* = 0.78
Duration of floaters symptoms	*p* = 0.16
Refraction	*p* = 0.78
Vitreous status	*p* = 0.40

* *p*-value was calculated with the Mann–Whitney U-Test.

## Data Availability

The raw data supporting the conclusions of this article will be made available by the authors on request.
